# Fenestrated Aortic Arch Endovascular Repair for Aortic Diseases Extending to Ishimaru Zones 2 and 3

**DOI:** 10.1177/15266028251324826

**Published:** 2025-03-18

**Authors:** Petroula Nana, Giuseppe Panuccio, José I. Torrealba, Fiona Rohlffs, Tilo Kölbel

**Affiliations:** 1 German Aortic Center, Department of Vascular Medicine, University Heart and Vascular Center UKE Hamburg, Hamburg, Germany; 2 Department of Vascular Surgery, University Hospital Regensburg, Regensburg, Germany

**Keywords:** endovascular aortic arch, distal arch, stroke, factors, LSA preservation

## Abstract

**Introduction:**

Fenestrated endovascular aortic arch repair (fTEVAR) has been successfully used for the exclusion of aortic lesions extending to distal arch. This study aimed to present the outcomes of fTEVAR for the preservation of the left common carotid artery (LCCA) or left subclavian artery (LSA) in lesions extending to Ishimaru zone 2 and 3.

**Materials and Methods:**

A single-center retrospective analysis of patients managed with fTEVAR for the preservation of the LCCA or LSA, between September 1st, 2011 and December 31st, 2023, was conducted, following the STROBE guidelines. Only preloaded fenestrated custom-made devices (Cook Medical, Bloomington, IN, USA) were used. Primary outcomes were technical success, mortality, and stroke at 30 days. Survival and freedom from secondary intervention were assessed using Kaplan–Meier estimates.

**Results:**

Seventy-five patients were included [72 years (IQR 13), range 48–86; 66.7% males]; 54 scheduled for LSA and 21 for LCCA preservation. Seven (9.3%) were treated urgently. Twenty-one (28.0%) presented with aortic dissection; 19 type B. Ishimaru zone 2 disease extension was recorded in 44 (58.7%) and zone 3 in 32 (42.7%). Debranching was performed in 22 patients: 81.8% LCCA-LSA bypass. Technical success was 93.3% with proximal landing to zone 0 in 18.7% cases, zone 1 in 70.7%, and zone 2 in 10.6%. Six (8.0%) deaths were recorded at 30-days and 4 (5.3%) strokes; 2 (2.7%) major. All strokes were diagnosed in patients with LCCA preservation. The multivariate analysis showed LCCA bridging (OR 0.2, 95% CI 0.08–0.3, p < 0.001) as independently related to stroke. The median follow-up was 12 months. The survival and freedom from secondary intervention were 85.2% [standard error (SE) 4.7%] and 75.0% (SE 6.5%) at 12 months, respectively.

**Conclusion:**

Patients treated by fTEVAR for diseases extending to zones 2 and 3 presented encouraging early outcomes. LCCA bridging seems to be independently related to higher stroke rate. Preservation of the LSA seems safe, without neurological consequences.

**Clinical Impact:**

Fenestrated endovascular arch repair has been applied with acceptable mortality in distal aortic arch lesions. However, the published experience is limited. This retrospective study of 75 patients with disease extend to zones 2 and 3 showed encouraging early outcomes with 93.3% technical success, 8.0% mortality, and 5.3% strokes. The inclusion of the left common carotid artery to the repair was related to higher stroke rate, while the preservation of the left subclavian seems to have no neurological consequences.

## Introduction

Recent data confirmed that the application of complex endovascular procedures for diseases affecting the aortic arch present satisfactory technical success and early mortality, but with a stroke rate at 10%.^1,2^ Landing proximal to Ishimaru zone 3 has been related to higher stroke rates.^3^ Sealing in the transverse arch with a stented fenestration or branch for the left subclavian artery (LSA) or left common carotid artery (LCCA) presents significant differences in terms of underlying disease, execution, and complications, compared to more proximal endovascular aortic arch repair sealing.^3,4^

Fenestrated endovascular aortic arch repair (fTEVAR) using preloaded custom-made devices (CMD) has been related to 4% 30-day mortality and 5.6% major stroke rate, showing that fTEVAR may provide a promising solution.^5–7^ Cervical debranching may be adjunctively performed during fTEVAR to provide an appropriate proximal landing zone; especially when a single fenestration device is used for the preservation of the LCCA. Recent data showed that additional debranching of the supra-aortic vessels, in conjunction to endovascular aortic arch repair, is a safe procedure, with low rates of cerebrovascular adverse events and failed patency rates.^8^ However, the published experience on fTEVAR is quite limited and further data is needed.^5,6,9^

The aim of this study was to present the early and midterm outcomes of fTEVAR using preloaded CMDs for the preservation of the LCCA or LSA in aortic lesions extending to zones 2 and 3 in a single high-volume aortic center.

## Materials and Methods

### Study Design

A single-center retrospective analysis of consecutive patients managed with fTEVAR using pre-loaded CMDs for the preservation of the LCCA and LSA, between September 1st, 2011 and December 31st, 2023, was conducted. The study period is representative of the total experience of the department, including patients of the learning curve up to the latest treated cases, to provide real world data and to decrease reporting bias. The STrengthening the Reporting of OBservational studies in Epidemiology (STROBE) guidelines (Supplemental Figure 1) were followed.^10^ The study complied with the Declaration of Helsinki and was considered exempt from ethical approval, given the retrospective nature and presentation of anonymized data, in accordance with the local state laws (§12 HmbKHG).

### Patient Population

All patients were treated, using fenestrated CMD relying on the Zenith platform (Cook Medical, Bloomington, IN, USA), in the same hybrid room under fluoroscopic fusion guidance. A single-device configuration was used in all cases, including a preloaded fenestration for the preservation of the target vessel (TV); LCCA or LSA and a proximal scallop for the preservation of the innominate artery (IA) or LCCA, respectively.^5^ In case that the fenestration was targeting the LCCA, a left common carotid-subclavian bypass or transposition was performed.^5^

Details on endograft characteristics and use of preloaded fenestrations have been reported previously.^5,8^ Patients were managed in supine position, with the left arm positioned at the side of the body with the palm facing forward. All patients were treated under general anesthesia, with systemic heparinization at 100 IU/kg, targeting an active clotting time at 250 to 350 seconds. Femoral access was used in all cases for main device introduction. Ultrasound-guided percutaneous femoral access was preferably used for main device advancement. Left percutaneous brachial access in the cubital fossa was used in all cases for establishing a through and through wire, using the preloaded catheter, between the femoral and brachial access, to secure device orientation and fenestration access.^5^ However, in 3 cases, an open cut-down of the LCCA was also used for stent advancement. No bailout transapical access was needed in any case. TVs preserved using scallops did not require bridging, except if device misalignment and compromise of TV’s flow was detected intra-operatively. The delivery systems were prepared by flushing with carbon dioxide (CO_2_), followed by 60 cc of saline.^11,12^

Patients with aortic arch, thoraco-abdominal, or descending thoracic aneurysms were included, as well as patients with penetrating aortic ulcers (PAU), and pseudoaneurysms, regardless of the underlying mechanism causing the disease (dissection or degenerative). Elective and urgent cases were included. Patients needing repair under urgent setting were managed using available CMDs.^13^ The criterion to perform fTEVAR under urgent circumstances was either that the patient was considered as high-risk for an open surgical repair (all had anamnesis of previous sternotomy) and a device from another patient with similar anatomy was available or were scheduled for an elective f/TEVAR, but symptoms evolution led to earlier treatment, using the device designed for this specific case. Patients selected for urgent repair using fTEVAR should present an adequate proximal aortic landing zone. Previously replaced proximal aortic landing zone and the absence of distal completion after fTEVAR was not a criterion for exclusion.

Proximal landing zone length ≥30 mm and diameter ≤38 mm were preferably chosen, especially for degenerative aneurysm cases while a compromise of proximal landing zone length to 20 to 25 mm could be selectively chosen in patients without degenerative aneurysm disease.^8^ All devices were oversized by 10% to 20% to the proximal aortic diameter; with 10% to 15% applied in dissection cases. Distal sealing was obtained either during fTEVAR or in a second stage (timespan ≥2 weeks) depending on patients’ anatomy.

### Target Vessels

TVs were catheterized using left brachial access after percutaneous ultrasound-guided puncture or LCCA cut down. Cervical debranching was performed, either with LCCA-LSA bypass or LSA transposition when LCCA bridging was planned.^14^ In case of aberrant right subclavian artery (RSA), a right common carotid artery (RCCA)-RSA bypass was performed. The aberrant RSA was then plugged (3 cases) while in case of endoleak type II, additional coil embolization could be performed. Balloon-expandable, covered stents were used for both the LCCA or LSA (Advanta V12; Getinge/Atrium Medical Corporation, Merrimack, NH, USA; BeGraft Peripheral, Innomed, Bentley, Hechingen and iCover, iVascular, Barcelona, Spain).^6^ Bridging stents were protruding about 4 mm into the main endografts’ lumen and were flared using 10 to 12 mm plain angioplasty balloons.

### Intraoperative Parameters

Regarding the methods to decrease the cardiac output during device deployment, up to December 2019, patients were managed using an inferior vena cava occlusion balloon, with a mean arterial pressure target at 30 mmHg or half the baseline value for a maximum 1 minute. Since January 2020, patients were managed with a combination of Valsalva maneuver and pharmacological management of the mean arterial pressure with a target mean arterial pressure half the baseline value.

Cerebrospinal fluid drainage (CSFD) was used prophylactically, for 48 to 72 hours postoperatively, in patients at high risk for spinal cord ischemia (SCI).^15^ As high-risk were considered patients presenting 2 or more of the following criteria: previous or simultaneous coverage >200 mm of the thoracic aorta or >40 mm proximal to celiac trunk, previous or simultaneous treatment extending to the abdominal aorta and unilateral or bilateral occlusion or high-grade stenosis of the internal iliac arteries. None of the patients of the current cohort presented signs of compromised LSA flow. Patients under therapeutic anticoagulation at the time of procedure or hemodynamically unstable were excluded from this protocol.^15^ Patients presenting symptoms attributable to SCI received a therapeutic CSFD.^15^ All cases were managed under near-infrared spectroscopy. Neither transesophageal echocardiography nor intravascular ultrasound represented standard practice intraoperatively. Extubation was routinely performed postoperatively in the hybrid operating room or within the initial postoperative hours. All patients were transferred to the intensive care unit for at least 24 hours. Patients presenting symptoms or signs that could be related to cerebral events (stroke, transient ischemic attack, or SCI) underwent evaluation by neurologists and further, adequate imaging with computed tomography or magnetic resonance imaging.

### Exclusion Criteria

Patients treated with physician-modified grafts, in situ fenestrations, or the parallel graft technique were excluded, as well as patients with branched devices of any type, including single-branch devices for the preservation of the LSA. Fenestrated devices with scallop or fenestration only were also excluded to increase the conformity of the cohort.

### Data Collection

Pre-, intra-, and postoperative data, after pseudonymization, were collected in a local database. Data collection was performed by experienced study nurses, in close collaboration with vascular surgeons. Regarding follow-up outcomes, the surveillance protocol included computed tomography angiography (CTA) before discharge, at 12 months, and yearly thereafter. Imaging surveillance could be modified, depending on CTA findings or in case of patients presenting symptoms. Available data on patient’s clinical status and imaging findings during follow-up were also added in the same database.

### Definitions

The lesions’ location within the different aortic segments was defined by the largest perpendicular aortic diameter setting the indication for repair. If the diameter of an aneurysm exceeded 55 mm within the aortic arch, this was classified as aortic arch aneurysm, regardless its proximal or distal extension. When the widest portion of the aneurysm was into the descending thoracic or thoracoabdominal aorta, the case was classified as thoracic aortic aneurysm or thoracoabdominal aortic aneurysm (TAAA). Arch type (type I, II, and III) was defined according to Madhwal et al.^16^ Bovine arch was considered the anatomic variation of a common IA and LCCA orifice.^17^ No true bovine arch (common orifice of all vessels) was recorded. The anatomic variation of a RSA stemming below the LSA and crossing the posterior mediastinum was considered as aberrant subclavian artery.^18^ No right-sided arch was included.

Urgent repair included patients with ruptured, symptomatic, and ≥80 mm diameter aneurysms (only when these patients were managed during the same hospitalization) or acute aortic dissection (only 1 case of type B aortic dissection with pleural effusion was managed during the acute phase).^13^

The Society for Vascular Surgery (SVS) reporting standards on the endovascular repair of aneurysms involving the reno-visceral aorta were used for definitions, due to lacking reporting standards for the endovascular repair of the aortic arch.^19^ Technical success was the composite of successful access to the arterial system, delivery and deployment of the main endograft and adjacent components to the intended site, side branch catheterization, and bridging stent deployment with patent intended TVs.^19^ Regarding types I and III endoleaks their presence in the completion angiography was considered a criterion for technical failure, only if the endoleak was still detected after the 30-day follow-up.^19^ Postoperative strokes, according to the modified Rankin scale (minor: Score 0–3 and major: Score 4–6) and SCI classification (Grades 1, 2, 3) were also recorded.^19^ Acute kidney injury was the reduction of the baseline glomerular function rate by >25% or any new onset dialysis after repair.^19^ According to the SVS reporting standards, secondary intervention was defined as any graft-related unplanned reintervention during follow-up. Scheduled procedures were not included as they represent stages of the index procedure.^19^

### Outcomes

Primary outcomes were the technical success, mortality, and stroke rates at 30 days. Survival and freedom from secondary interventions during follow-up were secondary outcomes.

### Statistical Analysis

Normally distributed continuous data were reported as mean ± standard deviation and nonnormally distributed as median values with range and IQR. Categorical data were expressed as absolute numbers and percentages. Chi-square test was used for categorical data comparison. Independent 2-sample *t* tests were used for normally distributed continuous variables, and the Mann–Whitney *U* test for for nonnormally distributed continuous and ordinal variables. Univariate and multivariate regression analyses were performed to investigate potential factors related to 30-day mortality, and stroke. Native proximal landing zone, early experience (including cases between 2011 and 2015), sex, underlying dissection, extend of the disease, and proximal landing in zones 0 and 1, type of lesion, type of arch, presence of bovine arch, urgent repair, LCCA revascularization were examined for all 3 primary outcomes. In addition, the use of CO_2_ and technical success were analyzed for stroke while technical success, stroke, SCI, and retrograde type A dissection for mortality. Only the factors detected as significant within the univariate analysis underwent multivariate logistic regression analysis (enter method). Kaplan–Meier estimates were performed to assess follow-up outcomes. No correction for multiple hypothesis testing was applied. The sample size was allowed to vary based on the analysis, and no imputation of missing data was performed, as for both categorical and continuous variables was infrequent (<5%). p Value was considered significant when it was <0.05 (2-tailed hypothesis). Statistical analysis was performed by SPSS 29.0 for MacOS Software (IBM Corp, Armonk, NY, USA).

## Results

In total, 75 patients were included. Among them, 54 fenestrations were planned for the LSA and 21 for the LCCA. The median age was 72 (IQR 13, range 48–86) years, and 66.7% were males. The baseline characteristics of the patients are presented in Table 1. The median American Score of Anesthesiologists (ASA) score was 3 (IQR 0, range 1–4) and 58 (77.3%) patients had ASA score ≥3.

**Table 1.** Baseline Characteristics of Patients Managed With Fenestrated Endovascular Aortic Arch Repair for Diseases Extending in Zone 2 and 3.

Variable (N, % or mean ± SD)	F-Arch cohort (75 patients)
Age (years)	70.8 ± 5.1
Males	50 (66.7)
Tobacco use	30 (40.0)
Active tobacco use	15 (20.0)
Hypertension	68 (90.7)
Diabetes mellitus	10 (13.3)
Dyslipidemia	36 (48.0)
Coronary artery disease	40 (53.3)
Myocardial infarction	8 (10.7)
CABG	6 (8.0)
Percutaneous coronary intervention	14 (18.7)
Chronic heart failure	14 (18.7)
COPD	12 (16.0)
Chronic kidney disease	10 (13.3)
Creatinine at admission (mg/dL)	1.1 ± 0.2
eGFR (mL/min)	68.1 ± 11.3
Dialysis	0 (0.0)
Past neurologic events	8 (10.7)
Stroke	6 (8.0)
Transient ischemic attack	2 (2.7)
Peripheral arterial disease	9 (12.0)

Abbreviations: CABG, coronary aortic bypass grafting; COPD, chronic obstructive pulmonary disease; eGFR, estimated glomerular filtration rate.

The median aortic diameter was 55.5 (IQR 14.5, range 35–90)mm. Seven (9.3%) patients were treated urgently: 4 (5.3%) due to symptomatic disease [3 aortic arch aneurysms and 1 type B aortic dissection (TBAD) due to pleural effusions] and 3 (4.0%) due to rupture (2 pseudoaneurysms and 1 PAU). Patients with underlying aortic dissection, except the previously mentioned TBAD case, were managed under elective setting. Twenty-one (28.0%) patients had a history of previous aortic repair of any segment (Table 2).

**Table 2.** Aortic History, Aortic Lesions’ Classification and Arch Type in Patients Treated With Fenestrated Endovascular Aortic Arch Repair for Lesions Extending to Zones 2 and 3.

Variable (N, %)	F-Arch cohort (75 patients)
Aortic history	
Ascending aortic replacement	1 (1.3)
Hemiarch replacement	5 (6.7)
Nonnative proximal landing zone	5 (6.7)
Aortic valve repair	2 (2.7)
Metallic aortic valve	1 (1.3)
TEVAR	8 (10.7)
f/bEVAR	1 (1.3)
EVAR	2 (2.7)
Open abdominal aortic repair	4 (5.3)
Aortic lesion	
Arch aneurysms	10 (13.3)
Degenerative	6 (8.0)
Postdissection	4 (5.3)
Penetrating aortic ulcer	22 (29.3)
Related to dissection	1 (1.3)
Pseudoaneurysms	9 (12.0)
Related to Kommerel’s diverticulum	3 (4.0)
TAAA types I and II	33 (44.0)
Degenerative	17 (22.7)
Postdissection	16 (21.3)
Thoracic aortic aneurysms	1 (1.3)
Degenerative	1 (1.3)
Postdissection	0 (0.0)
Arch type	
Type I	22 (29.3)
Type II	38 (50.7)
Type III	15 (20.0)

Abbreviations: EVAR, endovascular abdominal aortic repair; f/bEVAR, fenestrated and branched endovascular aortic repair; TAAA, thoracoabdominal aortic aneurysm; TEVAR, thoracic endovascular aortic repair.

Twenty-one (28.0%) patients had an underlying aortic dissection; 19 TBAD and 2 residual type A aortic dissections after hemiarch repair. The remaining 54 (72.0%) patients had degenerative disease. Thirty-three (44.0%) patients were managed for TAAA needing proximal extension of landing within the aortic arch and 22 (29.3%) were managed for PAU (Table 2). Nine patients were managed for pseudoaneurysms. No lesion extended proximally up to Ishimaru zone 1 or 0. Ishimaru zone 2 disease extension was recorded in 44 (58.7%) patients, and the remaining (32, 42.7%) presented with Ishimaru zone 3 extension of disease. Six (8.0%) patients presented bovine arch configuration while 3 had a Kommerel’s diverticulum and an aberrant RSA configuration.

### Operative Details

Debranching was performed in 22 (29.3%) patients. In 3 cases, a RCCA-RSA bypass was performed due to aberrant RSA (in one of the 3 cases, it was combined with LCCA-LSA bypass). The remaining cases were managed either with LCCA-LSA (18 patients, 81.8%) or LSA transposition (1 patient, 4.5%). All patients underwent debranching before the fTEVAR procedure, except 3 (13.6%), who were offered debranching during fTEVAR repair (2 due to transfer/organization reasons and 1 in the context of an urgent procedure). No stroke was recorded during or after debranching.

Technical success was 93.3% with 5 technical failures recorded: 1 due to endograft migration proximally (bailed-out with an in situ fenestration), 1 needing open conversion after retrograde type A dissection, 1 with type Ia endoleak persisting at 30-days, and 2 due to endograft malrotation (1 malrotation case was managed with triple parallel graft, while in the second malrotation case, the TV was finally bridged). One of the technical failures was recorded in a patient with aortic dissection, leading to a technical success rate for this subgroup at 95.3%. Table 3 provides details on TV bridging stents and relining. Proximal landing extended up to zone 0 in 14 (18.7%) cases, in zone 1 in 53 (70.7%), and in zone 2 in 8 (10.6%) cases. Forty (53.3%) patients received an additional distal thoracic extension intra-operatively; 7 (17.5%) with distal landing to zone 4, 24 (60.0%) with distal landing to zone 5, and the remaining (22.5%) to zone 6 (upper limit). False lumen endografts/Candy-Plugs were deployed in 10 cases of aortic dissection (47.6%, 10/21).

**Table 3.** Bridging Stent Distribution for the Preservation of the Left Common Carotid and Left Subclavian Artery During f-Arch in Patients With Diseases Involving Ishimaru Zones 2 and 3.

Bridging stents	Number of stents (%)
Innominate artery (1)	
Balloon expandable covered stents	1 (100)
Lifestream (Bard Medical, Tempe, AZ, USA)^a^	1 (100)
Left common carotid artery (22)	
Balloon expandable covered stents	22 (100)
iCover (iVascular, ES)	3 (13.7)
Lifestream (Bard Medical, Tempe, AZ, USA)	1 (4.5)
Advanta V12 (Getinge / Atrium Medical Corporation, Merrimack, NH)^b^	18 (81.8)
Relining^b^	9 (40.9)
Protégé Everflex (Medtronic, Santa Rosa, CA, USA)	9 (100)
Left subclavian artery (54)	
Balloon expandable covered stents (54)	54 (100)
BeGraft Peripheral (Innomed, Hechingen, GE)	1 (1.8)
iCover (iVascular, ES)	1 (1.8)
Lifestream (Bard Medical, Tempe, AZ, USA)	2 (3.6)
Advanta V12 (Getinge / Atrium Medical Corporation, Merrimack, NH)^b^	52 (92.7)
Relining^b^	10 (17.9)
Protégé Everflex (Medtronic, Santa Rosa, CA, USA)	10 (100)

a Bailout with chimney after endograft malrotation.

b One used during bailout with chimney after endograft malrotation.

Final angiography revealed 8 (10.7%) endoleaks, 5 (6.7%) were type Ia, 1 (1.4%) was type Ib (staged procedure), and 2 (2.7%) type II (one from an aberrant RSA managed with coiling intra-operatively). In one case of type Ia endoleak, additional endostapling was performed due to risk of endograft collapse. The median procedure time was 150 (IQR 92.5, range 66–640) minutes, with a median fluoroscopy time of 23.8 (IQR 12.5, range 7–93) minutes. The median volume of contrast was 109 (IQR 69, range 42–260) mL.

### Thirty-Day Outcomes

Six (8.0%) patients died during the initial 30-days; all deaths were in-hospital. Two patients died due to major stroke, 1 due to respiratory failure, and 3 due to retrograde type A dissection. Postoperative complications are presented in Table 4.

**Table 4.** Thirty-Day Postoperative Outcomes of Patients Treated With Fenestrated Endovascular Aortic Arch Repair for Diseases Extending to Zones 2 to 3.

Variable (N, %)	f-Arch cohort (75 patients)
Mortality	6 (8.0)
Stroke	4 (5.3)
Major stroke	2 (2.7)
Minor stroke	2 (2.7)
Spinal cord ischemia	4 (5.3)
Grade 1	2 (2.7)
Grade 2	2 (2.7)
Grade 3	0 (0.0)
Complete recovery	3 (4.0)
Late evolution	1 (1.3)
Cerebrospinal fluid drainage	11 (14.7)
Complications related to CSFD^a^	1 (9.0)
Prophylactic CSFD	8 (10.7)
Retrograde TAAD	3 (4.0)
Myocardial infarction	1 (1.4)
Congestive heart failure	2 (2.7)
Pericardial effusion	2 (2.7)
Acute kidney injury	1 (1.4)
Dialysis	0 (0.0)
Respiratory failure	2 (2.7)
Access complications	15 (20.0)
Lower access complications	13 (17.3)
Hematoma	5 (6.7)
Pseudoaneurysms	4 (5.3)
Ischemic complications	3 (4.0)
Infection	1 (1.4)
Upper extremity access complications	4 (5.3)
Pseudoaneurysms^b^	1 (1.4)
Hematoma	1 (1.4)
Ischemic complications^c^	2 (2.7)
Access-related reinterventions	5 (6.7)

Abbreviations: CSFD, cerebrospinal fluid drainage; TAAD, type A aortic dissection.

a Hematoma managed conservatively.

b One case of concomitant lower access related ischemia and pseudoaneurysm of the brachial artery.

c One case of concomitant lower access bleeding and thrombosis of the brachial artery.

Four (5.3%) patients presented stroke; 2 (2.7%) major and 2 minor. All strokes were recorded in patients managed for LCCA preservation. Two were ischemic and 2 presented signs of hemorrhagic conversion. Diffuse lesion distribution, with predominance to the left circulation in 3 patients, was reported while in 3 cases the posterior circulation was also affected. Two patients with stroke died within the 30-day follow-up. Details on patients with stroke are presented in Table 5.

**Table 5.** Anatomic Details on Patients With Stroke After fTEVAR.

Patients with stroke after fTEVAR	Early experience	LCCA preservation	LSA-LCCA bypass	Urgent repair	Type of arch	Bovine arch	Underlying aortic dissection	LCCA dissection	LCCA atheromatosis	Arch atherosclerosis	Arch wall thrombus
Patient 1	Yes (2014)	Yes	No	No	I	No	No	No	No	No	No
Patient 2	Yes (2015)	Yes	Yes	Yes	II	No	No	No	No	Mild (less than 25% of the circumference)	No
Patient 3	No (2020)	Yes	Yes	No	II	No	No	No	No	Moderate (25%–50% of the circumference)	Mild (less than 25% of the circumference)
Patient 4	No (2020)	Yes	Yes	No	II	No	Yes	No	Moderate (25%–50% of the circumference)	Mild (less than 25% of the circumference)	No

Abbreviations: fTEVAR, fenestrated thoracic endovascular aortic repair; LCCA, left common carotid artery; LSA, left subclavian artery.

In terms of SCI, 4 (5.3%) patients presented symptoms: 2 with Grade 1 and 2 with Grade 2. No Grade 3 (paraplegia) SCI was recorded. All SCI patients had received distal TEVAR extension with landing within zone 5. All evolved immediately after the procedure while one presented after the initial 24 hours after bleeding related to access. An urgent therapeutic CSFD was applied, with a targeted cerebrospinal pressure at 5 cmH_2_O and/or a maximum drainage volume of 30 mL/hour. The hemoglobin goal was set at 10 mg/dL, and the mean arterial pressure was maintained above 90 mmHg. No patient received steroids or mannitol. Three patients presented complete recovery (2 with Grade 1 and 1 with Grade 2), while 1 patient with Grade 2 remained stable. CSFD related complications were not recorded. Access complications were recorded in 15 (20.0%) patients with 5 (33.3%) needing interventions (Table 4).

### Follow-Up Findings

**Figure 1.**
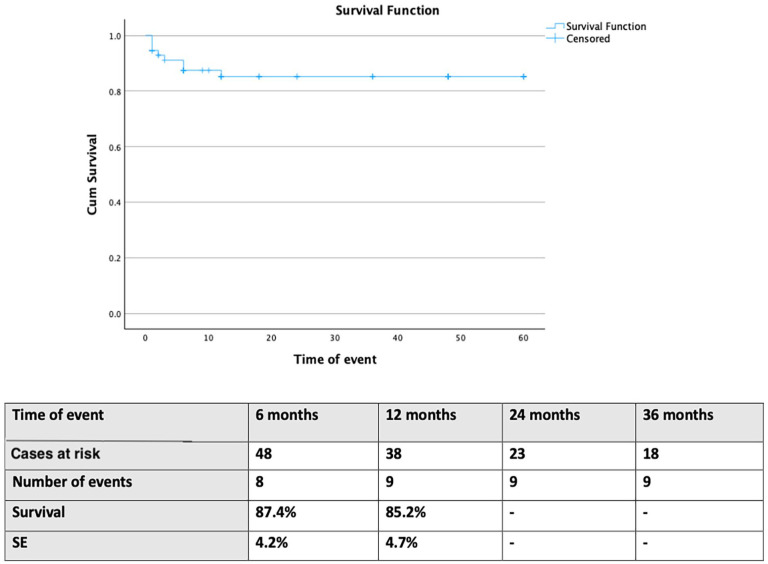
Follow-up estimated survival in 75 patients managed with fenestrated endovascular aortic arch repair for disease involving the distal arch. SE, standard error.

The median follow-up was 12 months (IQR 23, range 1–108 months). The estimated survival was 85.2% [standard error (SE) 4.7%] at 12 months, with all deaths being recorded within this first year of follow-up (Figure 1). Three deaths were aorta related (4.6%). Two patients died due to endograft infection: 1 at 2 months and 1 at 6-months postoperatively. The third patient presented with aortic rupture of a type II TAAA and was managed urgently with branch endovascular aortic repair (bEVAR). This patient died after evolving abdominal compartment syndrome.

**Figure 2.**
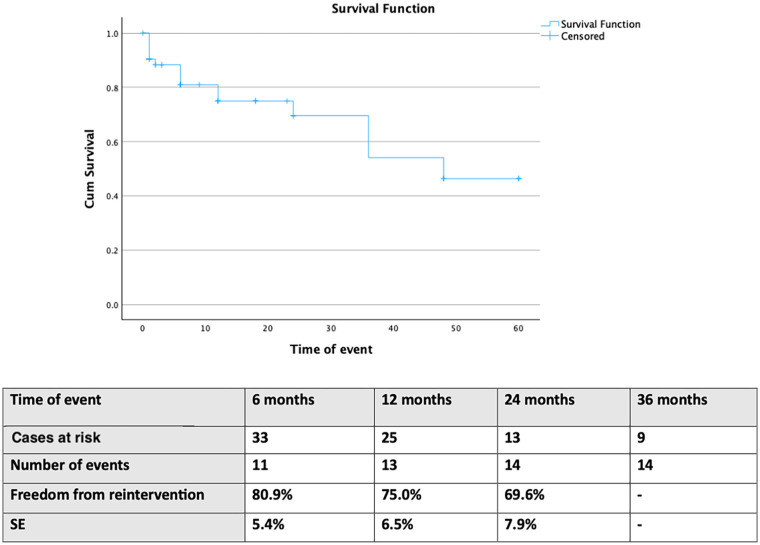
Follow-up freedom from secondary interventions in 75 patients managed with fenestrated endovascular aortic arch repair for disease involving the distal arch. SE: standard error.

The freedom from secondary interventions was 69.6% (SE 7.9%) at 24 months (in total 14 events; Figure 2). Four were classified as major secondary interventions: 2 patients were treated with an additional thoracic device for type IIIa endoleak, 1 patient was managed with bEVAR for an aortic rupture, and another was managed with open repair also for aortic rupture. Ten reinterventions were considered minor with 1 coiling for a type Ia endoleak, 1 LSA plug for type II endoleak and 8 of them being related to TV relining after signs of stent compression (5 LSA-stent relining, and 3 LCCA-stent relining). None of the minor reinterventions was related to clinical consequences. All stenosis events occurred in TVs bridged with the Advanta V12, which was used as bridging stent in 92% of TVs. TV primary-assisted patency was 100%. Freedom from TV stenosis was 77.6% (SE 7.6%) at 24 months (Supplemental Figure 2). In addition, 27 patients underwent secondary stages of management, leading to a freedom from any reintervention at 40.9% (SE 7.8%) at 24 months.

Freedom from any type of endoleak was 76.3% (SE 7.0%) at 24 months (Supplemental Figure 3). Among high-flow endoleaks, one type Ia endoleak persisted at 1-month follow-up. Six months later, the endoleak was managed with coil embolization of the false lumen. Two types of Ib endoleaks detected at 12-months were managed with distal endovascular repair (scheduled procedures), and 2 type IIIa were managed with TEVAR relining.

### Multivariate Analysis for Primary Outcomes

The univariate analysis for technical success identified only early experience as factor for technical failure. Multivariate analysis did not confirm this finding (p = 0.15). Mortality was related to technical success (p = 0.001), stroke (p = 0.001), and retrograde type A dissection (p < .001). The multivariate analysis confirmed all 3 factors: technical success (OR −0.18, 95% CI −0.42 to −0.01, p = 0.04), stroke (OR 0.18, 95% CI 0.01–0.42, p = 0.04), and retrograde type A dissection (OR 0.61, 95% CI 0.60–1.1, p < 0.001). The univariate analysis for stroke identified landing to zone 0 (p < 0.001) and 1 (p = 0.001), as well as the incorporation of LCCA for bridging (p < 0.001) but not early experience (p = 0.48). The multivariate analysis confirmed that LCCA bridging was an independent predictor for stroke (OR 0.2, 95% CI 0.08–0.3, p < 0.001). Aortic dissection or urgent repair were not detected as predictors for any adverse event in the univariate analysis and further investigation was not attempted.

## Discussion

The current study showed that fTEVAR for diseases affecting the distal arch can be used with high technical success, acceptable early mortality, and stroke rates. However, extended repair with LCCA incorporation to treatment increased the risk of stroke, at 19% within the LCCA cohort. Previous data showed that extending of proximal landing up to zone 2 with preservation of the LSA could be considered safe in terms of peri-operative cerebrovascular events.^3,20^ However, more proximal landing is followed by higher mortality and stroke rates.^3^ The findings of this analysis are in accordance with previous studies reporting on CMD fenestrated devices with mortality rates at 3.7% and major stroke at 5.6%.^5^

Technical success was high. Previous data showed high technical success rates, up to 99%, despite intra-operative complications, as wire entanglement.^5^ Endograft misalignment, under the use of the through-and-through wire, was the reason leading to failure in 2 out of 5 failed cases.^5^ Despite that final angiography confirmed the presence of 5 Ia endoleaks, at 30-days only one of them was present. This highlights the importance of a “wait and watch” approach of endoleaks in complex aortic endovascular repair, as reinterventions in such proximal zones are quite complex and cannot be always considered as harmless.^19^

Early mortality was 8% at 30 days and was independently related to technical failure, stroke, and retrograde type A dissection. Encouraging findings have been also reported from single branch devices targeting the preservation of the LSA, with null early mortality rate.^20–22^ Studies reporting on LSA debranching and standard TEVAR to extend proximal landing showed similar mortality rates, ranging from 0% to 10%.^23–25^ Despite the nondetected statistical significance, potentially attributed to the small sample size, parameters as urgent repair could also lead to worse outcomes in terms of mortality. Urgent management has been previously reported in the literature as a factor of increased mortality in patients managed with fenestrated and branched thoracic endovascular aortic repair (f/bTEVAR).^26^ Retrograde type A dissection is rather a fatal complication, mainly associated to native landing zones.^26–28^ In this cohort, all patients presenting retrograde type A dissection died within the early follow-up.

Branched and fenestrated devices perform similarly in terms of stroke, with rates up to 10% during the early postoperative period.^27^ Proximal landing to Ishimaru zones 0 and 1, in addition to native proximal aortic landing are factors leading to worse stroke outcomes.^26,27,29^ In the current cohort, strokes evolved only in patients needing more extensive repair with bridging of the LCCA, confirming that the proximity of landing and incorporation of carotid arteries play a significant role in stroke evolution while early experience was not detected as a predictor for stroke events.^29^ The diffuse distribution of the ischemic lesions in the postoperative cerebral imaging, should be acknowledged and potentially signifies that intra-operative fTEVAR stroke may be a multifactorial phenomenon, depending on anatomic and technical parameters, such as wire and device manipulations within the aortic lumen. Despite the lack of statistical significance of early experience on stroke rates, the evolution on device design through time, with the fixation of the free struts and edges of the scallop on the dilator, to prevent wire entanglement, and the improvement of device alignment using spiral wire attachment and a curved self-orientating delivery system, seem to have improved fTEVAR outcomes.^7^ Cervical debranching was not related to stroke in this cohort; a fact aligning with the recent literature reporting null stroke and high patency rates among patients receiving a combination of f/bTEVAR and supra-aortic trunk debranching, especially LCCA-LSA bypass.^9^ In terms of cerebrovascular complications, the SCI rate was estimated at 5.3%, despite that 44% of cases were managed for thoracoabdominal aneurysms. Staged approached and selectively applied CSFD probably led to acceptable rates of SCI, while no complications or deaths were related with the use of CSFD.^30^

Follow-up outcomes showed an 85.2% estimated survival at 12 months, while 3 deaths during the same period were related to aortic complications. Previous studies showed similar survival rates, lower than 80% during the initial 2 post-operative years.^5,6^ Despite encouraging early findings, midterm survival outcomes are rather discouraging and highlight the high-risk baseline profile of the patients. In addition, it should be noted that most patients were managed for extensive thoracoabdominal aortic disease, rather than localized aortic arch lesions. Under the light of previously published data, the estimated mortality rate should not be considered surprising.^31^ Patients managed with combined fenestrated-branched endovascular repair, extending from the aortic arch to the thoracoabdominal aorta, presented an estimated survival at 72% during the 12-month follow-up.^31^

Multiple reinterventions for endovascular aortic repair completion may be another reason leading to lower survival rates during follow-up. In this analysis, almost 60% of patients needed a reintervention, with 73% of them being scheduled. Previous multicenter data on patients managed with fTEVAR reported a freedom from reintervention at 73.0% during the mid-term follow-up, with a concomitant TV reintervention rate at 4%.^5^ Staged procedures are common within patients with extensive disease and provide a significant benefit for SCI prevention; as in the current cohort.^31^ Unplanned reinterventions were mainly attributed in this study to bridging stent compression. Despite high TV patency, adverse events of, mainly LSA, bridging stent compression may be underreported, as they are not always related to clinical consequences.^32^ The large diameter of the TVs, and subsequent bridging stents, in addition to relining may prevent stent occlusion and decrease the risk of severe clinical complications.^32^ In the current analysis, LCCA bridging stent relining rate was over 40%, this aggressive policy on LCCA relining has also been previously reported and may have been related to surgeon bias, tending to secure carotid artery patency in order to decrease potential future stroke events.^33^ Previous analyses showed that anatomic parameters related to the anatomy of the LSA, including the nearest bone structure and higher tortuosity were related to subclinical stent compression.^32^ However, further studies on the potential anatomic and technical characteristics affecting TV stent fate are needed.^33^

Alternatively, single bTEVAR has also been used to provide adequate proximal aortic landing in diseases extending in zone 2. The findings in the literature regarding the use of off-the-shelf bTEVAR devices are very encouraging with high technical success and low 30-day mortality.^34^ Regarding strokes, the estimated 30-day rate was at 5%, with all events being related to debranching procedures.^34^ All TVs remained patent during the initial 6 months of follow-up.^34^ Previous data on bTEVAR showed that the risk for TV-related endoleaks was around 10% at 24 months of follow-up, compared to null into the current analysis, but with the cost of asymptomatic bridging stent stenosis.^35^ Appropriate patient selection is of major importance while further studies focusing on the factors leading to adverse events in both approaches may assist better patient stratification.

## Limitations

The retrospective design, sample size, and patient selection bias could be considered the main limitations of the analysis. A variety of aortic arch lesions were managed; however, using the same device in all cases while all TVs were bridged using balloon-expandable covered stents increasing the conformity of the cohort. A selection of patients on lesion type, especially the exclusion of aortic dissection cases, would decrease even more the power of the analysis while it could potentially introduce reporting bias. However, the potential impact of dissection on the main outcomes of the analysis was investigated through a uni- and multivariate analysis, and no statistical significance was found. Comparisons between different stent types were not attempted as the vast majority of TV were bridged using the Advanta V12 (92%) and subsequently, all events occurred in this subgroup. Further comparative analyses on the use of bridging balloon-expandable covered stents may be able to identify benefits and pitfalls on the optimal stent selection. Patients presenting with silent cerebral lesions as well as the presence of shaggy aorta and aortic arch atherosclerosis were not analyzed in terms of postoperative stroke. The impact of stent diameter and relining on stroke were not examined. Selection bias should be acknowledged, as the number of patients rejected for fTEVAR was not available. Long-term outcomes are missing and should be attributed to the 38% loss to follow-up. The high rate of loss to follow-up has potentially affected outcomes and limited the reliability of Kaplan–Meier curves to 36 months (SE below 10%). However, such a loss to follow-up rate is not uncommon among patients managed with complex endovascular aortic repair and has been previously related to patient’s fatigue, social and economic parameters, and health system organization.^36,37^ Future studies may be able to shed more light on long-term outcomes and provide more robust conclusions on midterm findings. All outcomes should be interpreted cautiously under potential type II statistical error.

## Conclusion

Patients treated by fTEVAR for diseases extending to zones 2 and 3 present high technical success and acceptable early mortality, and major stroke rates. Taking into consideration the limitations of the study, the proximity of repair with LCCA bridging seem to be related to stroke during the postoperative period. Preservation of the LSA using fTEVAR may be a safe solution without neurological consequences.

## Supplemental Material

sj-docx-1-jet-10.1177_15266028251324826 – Supplemental material for Fenestrated Aortic Arch Endovascular Repair for Aortic Diseases Extending to Ishimaru Zones 2 and 3 Supplemental material, sj-docx-1-jet-10.1177_15266028251324826 for Fenestrated Aortic Arch Endovascular Repair for Aortic Diseases Extending to Ishimaru Zones 2 and 3 by Petroula Nana, Giuseppe Panuccio, José I. Torrealba, Fiona Rohlffs and Tilo Kölbel in Journal of Endovascular Therapy

sj-tiff-2-jet-10.1177_15266028251324826 – Supplemental material for Fenestrated Aortic Arch Endovascular Repair for Aortic Diseases Extending to Ishimaru Zones 2 and 3 Supplemental material, sj-tiff-2-jet-10.1177_15266028251324826 for Fenestrated Aortic Arch Endovascular Repair for Aortic Diseases Extending to Ishimaru Zones 2 and 3 by Petroula Nana, Giuseppe Panuccio, José I. Torrealba, Fiona Rohlffs and Tilo Kölbel in Journal of Endovascular Therapy

sj-tiff-3-jet-10.1177_15266028251324826 – Supplemental material for Fenestrated Aortic Arch Endovascular Repair for Aortic Diseases Extending to Ishimaru Zones 2 and 3 Supplemental material, sj-tiff-3-jet-10.1177_15266028251324826 for Fenestrated Aortic Arch Endovascular Repair for Aortic Diseases Extending to Ishimaru Zones 2 and 3 by Petroula Nana, Giuseppe Panuccio, José I. Torrealba, Fiona Rohlffs and Tilo Kölbel in Journal of Endovascular Therapy

sj-tiff-4-jet-10.1177_15266028251324826 – Supplemental material for Fenestrated Aortic Arch Endovascular Repair for Aortic Diseases Extending to Ishimaru Zones 2 and 3 Supplemental material, sj-tiff-4-jet-10.1177_15266028251324826 for Fenestrated Aortic Arch Endovascular Repair for Aortic Diseases Extending to Ishimaru Zones 2 and 3 by Petroula Nana, Giuseppe Panuccio, José I. Torrealba, Fiona Rohlffs and Tilo Kölbel in Journal of Endovascular Therapy
