# Supplementary material for: Fenestrated Aortic Arch Endovascular Repair for Aortic Diseases Extending to Ishimaru Zones 2 and 3
Source: J Endovasc Ther. 2025 Mar 18;33(4):2003–14. doi: 10.1177/15266028251324826 (PMC13371152; doi:10.1177/15266028251324826)
Supplement: sj-docx-1-jet-10.1177_15266028251324826 – Supplemental material for Fenestrated Aortic Arch Endovascular Repair for Aortic Diseases Extending to Ishimaru Zones 2 and 3 [file sj-docx-1-jet-10.1177_15266028251324826.docx]

**Supplementary Figure legends**

**Supplementary Figure 1.** The STROBE guidelines were followed for the conduction of the study. Only patients managed with fenestrated devices presenting a single fenestration and a proximal scallop were considered eligible.

**Supplementary Figure 2.** Estimated freedom from TV stenosis in 75 patients managed with fenestrated endovascular aortic arch repair for disease involving the distal arch. Footnotes: SE: standard error.

**Supplementary Figure 3.** Estimated freedom from endoleak in 75 patients managed with fenestrated endovascular aortic arch repair for disease involving the distal arch. Footnotes: SE: standard error.
