# Supplementary figures and images for: Fenestrated Aortic Arch Endovascular Repair for Aortic Diseases Extending to Ishimaru Zones 2 and 3
Source: J Endovasc Ther. 2025 Mar 18;33(4):2003–14. doi: 10.1177/15266028251324826 (PMC13371152; doi:10.1177/15266028251324826)

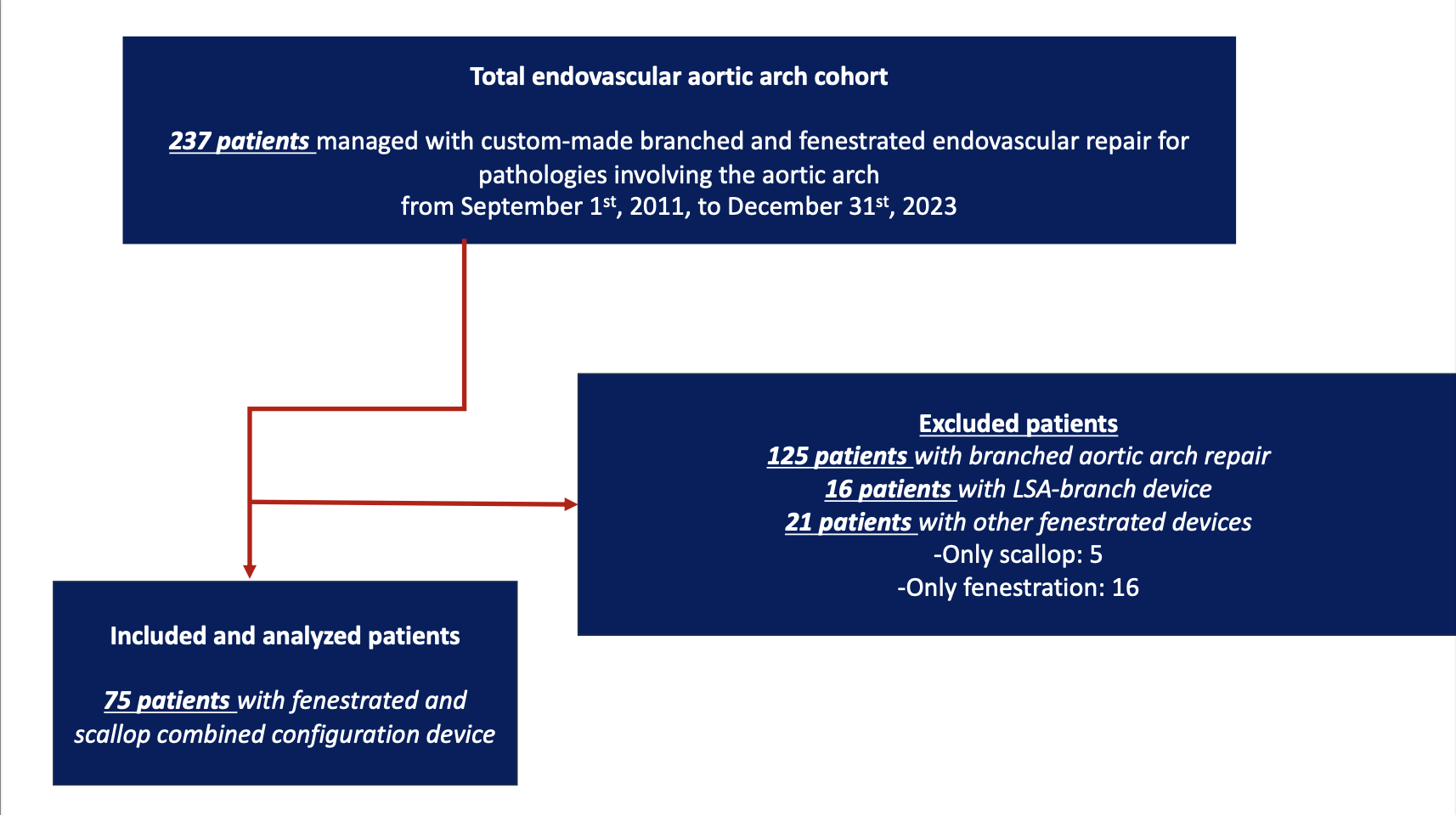

Supplement: sj-tiff-2-jet-10.1177_15266028251324826 – Supplemental material for Fenestrated Aortic Arch Endovascular Repair for Aortic Diseases Extending to Ishimaru Zones 2 and 3 [file sj-tiff-2-jet-10.1177_15266028251324826.tiff]

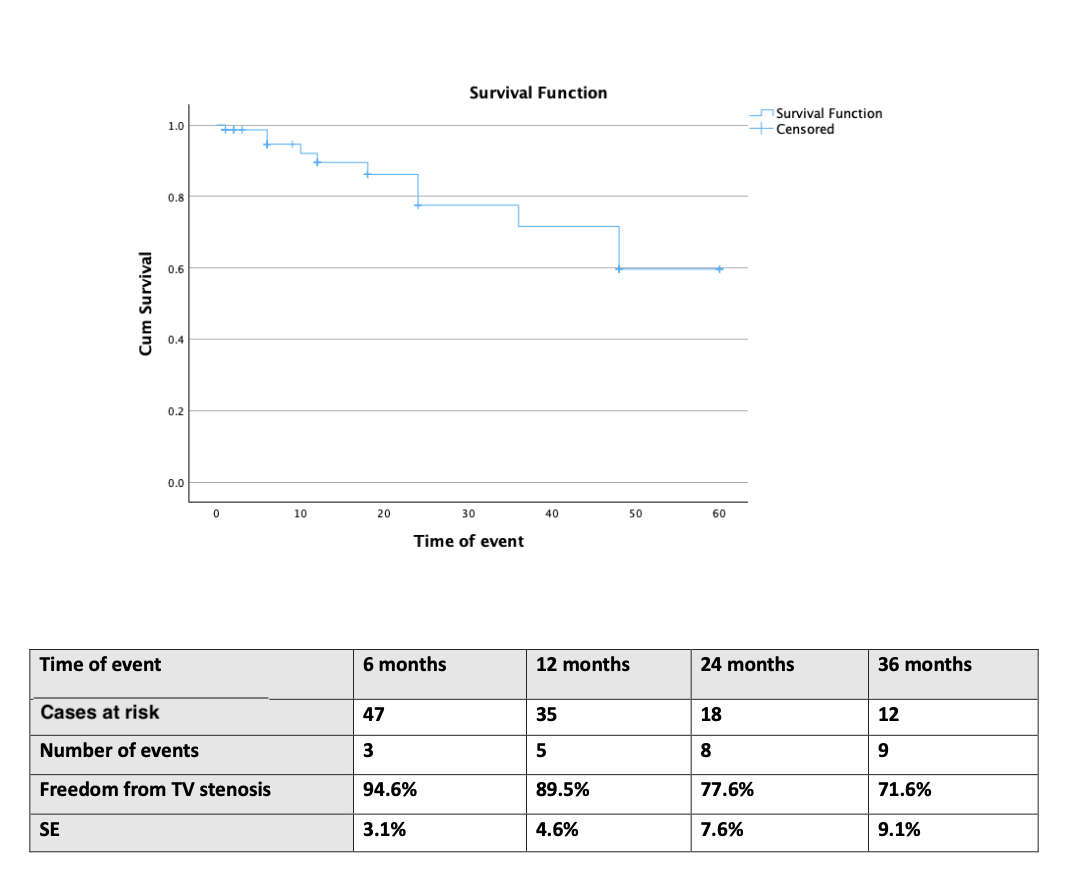

Supplement: sj-tiff-3-jet-10.1177_15266028251324826 – Supplemental material for Fenestrated Aortic Arch Endovascular Repair for Aortic Diseases Extending to Ishimaru Zones 2 and 3 [file sj-tiff-3-jet-10.1177_15266028251324826.tiff]

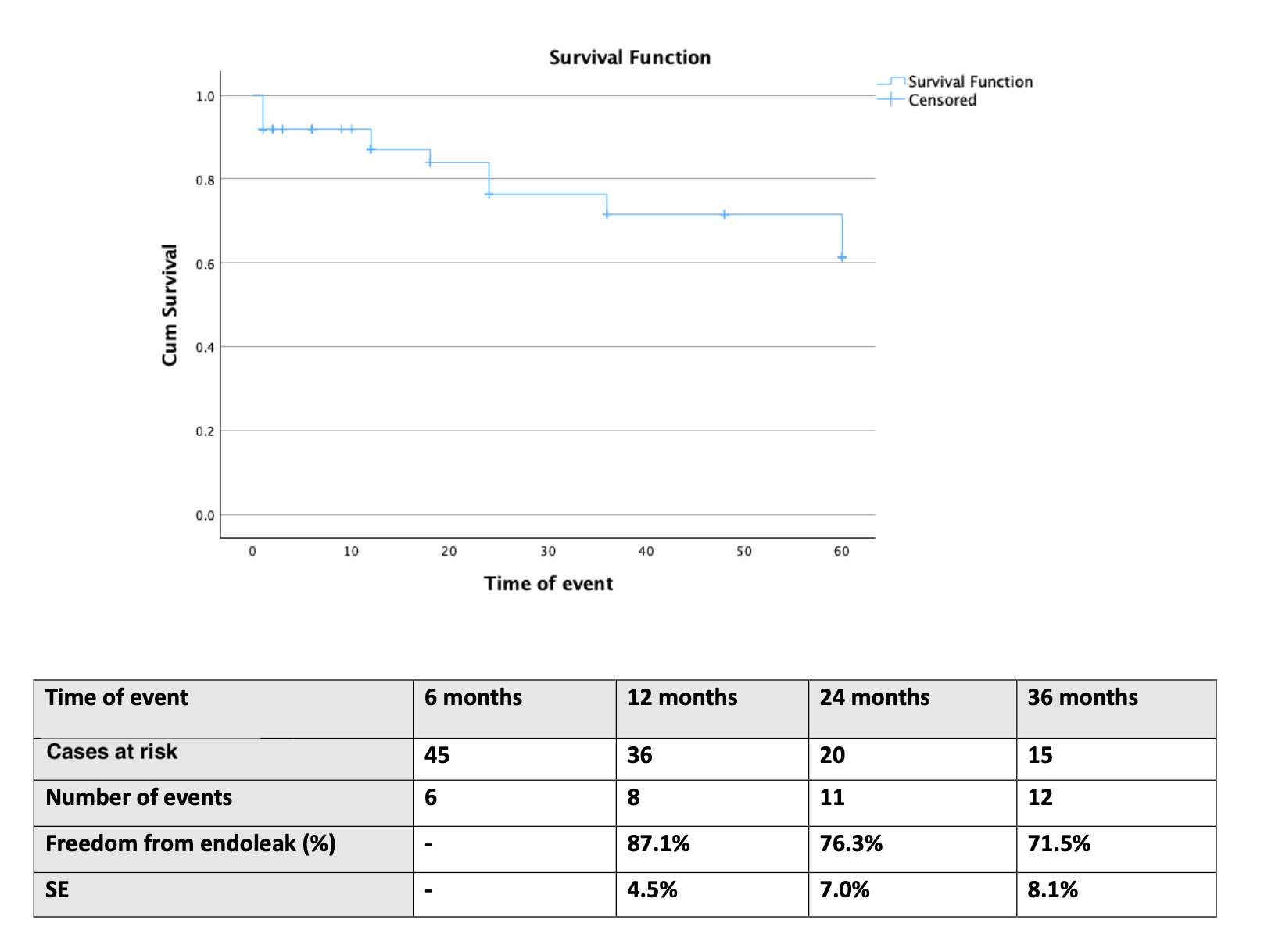

Supplement: sj-tiff-4-jet-10.1177_15266028251324826 – Supplemental material for Fenestrated Aortic Arch Endovascular Repair for Aortic Diseases Extending to Ishimaru Zones 2 and 3 [file sj-tiff-4-jet-10.1177_15266028251324826.tiff]
